# Wheat Long Noncoding RNAs from Organelle and Nuclear Genomes Carry Conserved microRNA Precursors Which May Together Comprise Intricate Networks in Insect Responses

**DOI:** 10.3390/ijms24032226

**Published:** 2023-01-23

**Authors:** Bala Ani Akpinar, Tugdem Muslu, Gadi V. P. Reddy, Munevver Dogramaci, Hikmet Budak

**Affiliations:** 1 Montana BioAgriculture, Inc., Missoula, MT 59802, USA; 2 USDA-ARS, Southern Insect Management Research Unit, Stoneville, MS 38776, USA; 3 USDA-ARS, Edward T. Schafer Agricultural Research Center, Fargo, ND 58102, USA

**Keywords:** long noncoding RNAs, gene regulation, wheat, microRNA, stress response, insect resistance, OWBM, WSS, target mimicry

## Abstract

Long noncoding RNAs (lncRNAs) are a diverse class of noncoding RNAs that are typically longer than 200 nucleotides but lack coding potentials. Advances in deep sequencing technologies enabled a better exploration of this type of noncoding transcripts. The poor sequence conservation, however, complicates the identification and annotation of lncRNAs at a large scale. Wheat is among the leading food staples worldwide whose production is threatened by both biotic and abiotic stressors. Here, we identified putative lncRNAs from durum wheat varieties that differ in stem solidness, a major source of defense against wheat stem sawfly, a devastating insect pest. We also analyzed and annotated lncRNAs from two bread wheat varieties, resistant and susceptible to another destructive pest, orange wheat blossom midge, with and without infestation. Several putative lncRNAs contained potential precursor sequences and/or target regions for microRNAs, another type of regulatory noncoding RNAs, which may indicate functional networks. Interestingly, in contrast to lncRNAs themselves, microRNAs with potential precursors within the lncRNA sequences appeared to be highly conserved at the sequence and family levels. We also observed a few putative lncRNAs that have perfect to near-perfect matches to organellar genomes, supporting the recent observations that organellar genomes may contribute to the noncoding transcript pool of the cell.

## 1. Introduction

Up to 90% of genomes are transcribed, throughout different stages of growth and development, of which only a small percentage of RNA transcripts are translated into proteins. The remaining regions lack protein-coding ability, causing these parts to be long considered “junk” DNA for a long time [1,2]. Advances in sequencing technologies improved our understanding of noncoding RNAs (ncRNAs) and have shown that cells transcribe their non-protein-coding DNAs into various types of RNA molecules, some of which regulate the expression of other genes on many levels [3]. Although the interest in ncRNA research has accelerated in the last two decades, the biological functions of many ncRNAs are still yet to be discovered.

Long noncoding RNAs (lncRNAs) are a class of ncRNAs that are typically longer than 200 nucleotides [4]. While lncRNAs structurally and functionally resemble messenger RNAs (mRNAs), lncRNAs are less stable, less abundant, less conserved, and they do not possess coding potential [5,6]. mRNAs are transcribed by RNA polymerase II (Pol II), while plant lncRNAs can be transcribed by one of the four DNA-dependent RNA polymerases, Pol II, Pol III, Pol IV, and Pol V [4,7,8]. The conservation of lncRNA transcripts among species is poor, and their complex secondary and tertiary structures make large-scale identification of lncRNAs and their targets challenging [4,9]. Therefore, lncRNA identification typically involves elimination of transcripts with known mRNA and small ncRNA characteristics [2,4,10]. Together with the aid of deep sequencing, the involvement of many lncRNAs in gene expression and remodeling eukaryotic genomes has been revealed [11]. Multiple lncRNA databases such as ﻿Plant Long NonCoding RNA Database version 2.0 [12] and NONCODEv6 [13] hold tens of thousands of plant lncRNAs, shown to contribute to the regulation of various developmental processes including growth, response to diseases, and abiotic stresses. However, the low sequence conservation of lncRNAs complicates comparative annotations on the basis of sequence homology with known lncRNAs [14].

In plants, lncRNAs regulate gene expression mostly through transcriptional regulation and alternative splicing and are usually expressed in a tissue-specific manner [4]. The mRNA-like characteristics of lncRNAs provide a way of indirect regulation involving microRNAs (miRNAs), another class of small, regulatory ncRNAs, in which “competitive endogenous” lncRNAs mimic mRNA targets of miRNAs [15]. To date, many lncRNAs from different plant species have been shown to participate in competitive endogenous RNA regulation. In rice, lncRNA TCONS_00021861 transcript has been shown to be the positive regulator of a drought-related gene, YUCCA7, through interacting with miR528 [16]. Another recent study on soybean validated two miRNA-lncRNA pairs, miR166-Gmax_MSTRG. 35921.1 and miR394-Gmax_MSTRG. 18616.1, which have antagonistic expression patterns upon salt treatment [17]. Besides being “miRNA baits”, lncRNAs can also act as precursors for other small ncRNAs, including miRNAs and small-interfering RNAs (siRNAs) [7]. LncRNAs that act as miRNA precursors have also been shown to function in critical biological processes, such as plant–pathogen interactions [18]. The dual functions of lncRNAs, being both miRNA regulators and miRNA sources, show the importance of unraveling lncRNA–miRNA relationships for a better understanding of gene expression regulation.

As a major source of energy, wheat is one of the most important crop species worldwide. Insect pests are a great challenge against meeting the current and future demands of wheat production. Wheat Stem Sawfly (*Cephus cinctus* Norton, WSS) and Orange Wheat Blossom Midge (also known as wheat midge; *Sitodiplosis mosellana*, OWBM) are two of the highly destructive, yield depriving insects pests of wheat [19,20]. Stem solidness, which restricts the larval growth inside the stems, has been the most widely utilized strategy in WSS management. Recently, the causal gene for WSS resistance, residing in a major quantitative trait locus (QTL) on the wheat 3B chromosome, has been identified [21]. This QTL remains the major source of WSS resistance and is still under scrutiny for both coding and noncoding features [19,20]. Similarly, a candidate gene has been recently proposed, using multiple wheat genomes, that provides resistance against OWBM that is located on the wheat 2B chromosome [22]. Despite severe efforts towards the identification of coding sequences that provide resistance against these devastating pests, noncoding transcripts that may also participate in the insect resistance response have not yet attracted much attention even though wheat lncRNAs have been linked to plant development and stress responses [14,23,24]. Here in this study, we aim to uncover the wheat lncRNA repertoire related to insect response, using transcriptome data from OWBM-resistant and susceptible bread wheat genotypes LX99 and 6128 with or without OWBM infestation, as well as durum wheat varieties CDC Fortitude, Kofa, Langdon, LDN-GB-3B, Pithless, and W9262-2603D that differ in their solid stemness and, hence, WSS resistance.

## 2. Results

### 2.1. Different Wheat Genotypes May Contain Several Lineage-Specific lncRNAs

A tiered approach was used to identify long noncoding RNAs (lncRNAs) in durum and bread wheat varieties, in which sequences not meeting previously established lncRNA criteria [4] were progressively removed. In tetraploid durum wheat, RNA-Sequencing (RNA-Seq) data from stem tissues of hollow-stemmed varieties Kofa, Langdon, Pithless and solid-stemmed varieties CDC Fortitude, LDN-GB-3B, and W9262-260D3 [21] yielded 160k–173k assembled transcripts (Table 1). Removal of sequences shorter than 200 nucleotides and sequences matching other types of noncoding RNAs, including tRNAs, rRNAs, small nuclear RNAs (snRNAs), and small nucleolar RNAs (snoRNAs), retained >150 k transcripts for each sample. Next, removal of transcripts that may contain open reading frames (ORFs) longer than 100 amino acids eliminated ~65% of the transcripts, likely corresponding to protein-coding sequences. For the remaining candidates, two different software, CPAT and CPC2, were used to calculate coding potentials, which were largely in agreement (Table 1). Transcripts that did not exhibit coding potentials in both predictions were finally compared to coding sequences from the reference *Triticum durum* Svevo genome. In this last step, transcript sequences that did not match any coding sequences were accepted as putative ‘clean’ lncRNAs, representing 21–22% of all the assembled transcripts (Table 1).

In the hexaploid bread wheat, RNA-Seq data from kernels of susceptible 6218 and resistant LX99 varieties, with or without infestation with the devastating wheat pest, Orange Wheat Blossom Midge (OWBM) [25], yielded 202–219k transcripts (Table 1). Similar to the durum varieties, the majority of the sequences (~60–65%) were eliminated for carrying predicted ORFs larger than 100 amino acids (Table 1). For coding potentials, predictions by CPAT and CPC2 were highly in agreement, retaining 62k–80k candidates for the final step. Comparing the remaining transcripts to high-confidence coding sequences from the reference *Triticum aestivum* Chinese Spring genome finally identified 42 k–57 k putative ‘clean’ lncRNAs, representing 21–26% of all assembled transcripts. Intriguingly, the number of putative lncRNAs, as well as their ratios to the overall transcripts, appear to decrease in the susceptible 6218 but increase in the resistant LX99 upon infection with OWBM (Table 1).

**Figure 1:**
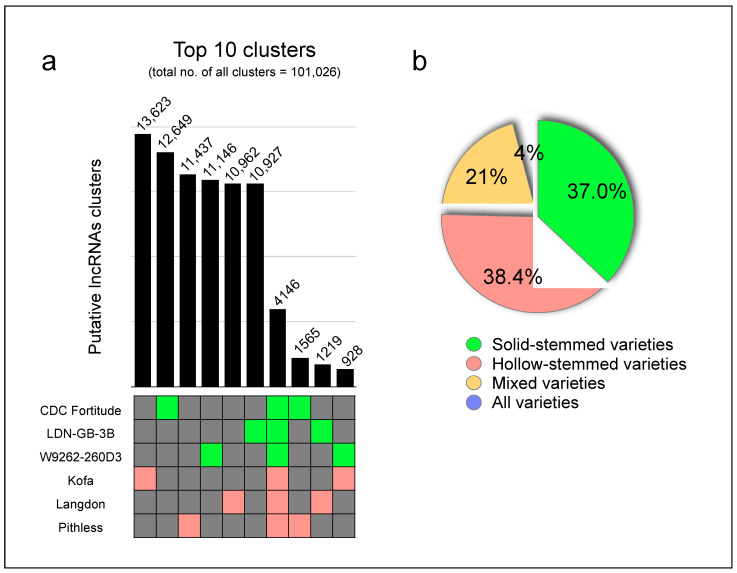
Clusters of durum wheat lncRNAs that share at least 90% sequence similarity. (**a**) The number of clusters that contain lncRNAs from one or more varieties (colored boxes) that do not share significant similarities with lncRNAs from other varieties (gray boxes). Boxes colored green, representing solid-stemmed varieties, and peach, representing hollow-stemmed varieties, indicate the presence of lncRNAs belonging to that variety for the given dataset. Boxes colored gray indicate absence. The numbers over the bars indicate the number of clusters for a given dataset. Only the top 10 clusters are given for simplicity. The full graph is given as Supplementary Figure S1. (**b**) The percentages of clusters that contain lncRNAs from only solid-stemmed varieties (green), only hollow-stemmed varieties (peach), all varieties included (purple), and the rest (yellow, mix of solid and hollow-stemmed varieties).

**Figure 2:**
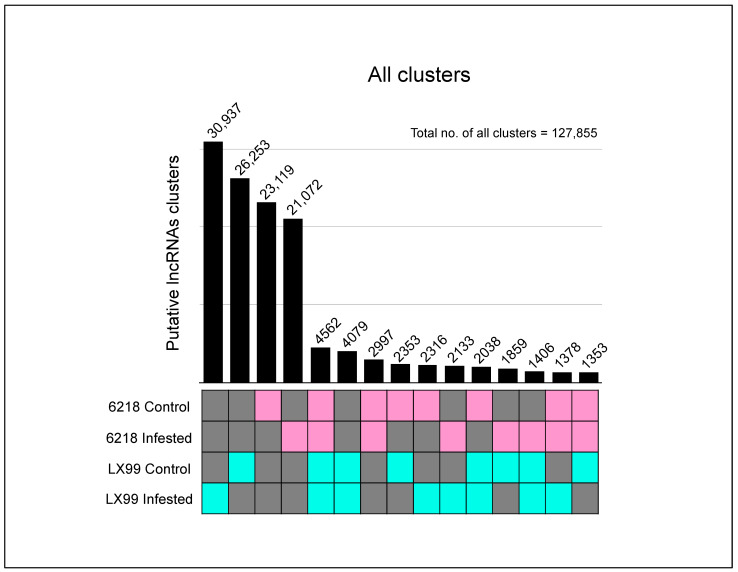
Clusters of bread wheat lncRNAs that share at least 90% sequence similarity. The number of clusters that contain lncRNAs from one or more samples (colored boxes) that do not share significant similarities with lncRNAs from other samples (gray boxes). Boxes colored pink, representing the susceptible 6218 variety, and blue, representing the resistant LX99 variety, indicate the presence of lncRNAs belonging to that variety for the given dataset. Boxes colored gray indicate absence. The numbers over the bars indicate the number of clusters for a given dataset.

A complicating factor in comparative identification and annotation of lncRNAs across different species and conditions is the poor level of conservation at the primary sequence level. To overcome this complication in comparing lncRNAs across different samples, putative lncRNAs from durum and wheat varieties were combined into clusters, separately for each species, that share at least 90% sequence identity. Due to the observed high sequence conservation at the sub-species level [26], each cluster arguably represents a single lncRNA and cluster members correspond to the same lncRNA in different varieties; in that sense, clusters can be viewed as ‘lncRNA families’. Clusters with members only from a single variety, thus, may represent variety or condition-specific lncRNAs. As seen in Figure 1 and Figure 2, most putative lncRNAs appear to be variety and/or condition-specific for both durum and bread wheat, even at the liberal 90% sequence identity (allowing up to 10 mismatches every 100 nucleotides). For durum wheat, each variety had more than 10 k clusters made up entirely of their own lncRNAs, without any significant similarities with lncRNAs from any other variety. Kofa had the largest number of self-specific clusters at over 13 k (Figure 1a, Supplementary Figure S1). In total, 4146 clusters had lncRNAs from all varieties, likely corresponding to homologous lncRNAs. Notably, clusters made up from lncRNAs from only hollow-stemmed or only solid-stemmed varieties made two large groups, representing 38.4% and 37% of all clusters, respectively (Figure 1b). Only about one fifth of all clusters had lncRNAs from both solid and hollow-stemmed varieties, excluding the clusters which had lncRNAs from all varieties included (4%). For bread wheat varieties, clusters with lncRNAs only from a given condition ranked at the top (Figure 2). Specifically, LX99 with infestation had almost 31k clusters that were composed of lncRNAs that did not have any significant similarities from other conditions, including LX99 lncRNAs without infestation (Figure 2). Similar to observations from durum wheat varieties, 4562 clusters contained lncRNAs from all control and infested samples, ranking fifth, right after clusters arguably specific to each sample. These observations suggest that many lncRNAs may be variety and/or condition specific, while lncRNAs that are potentially homologous across all varieties and/or conditions also form a sizable group. Even though clusters of lncRNAs are helpful in exploring conservation across different samples, due to a lack of certainty in actual conservation rates of lncRNAs at the sequence level so far, their use beyond comparative purposes may be problematic. Therefore, we refrained from using consensus lncRNA sequences from cluster representatives, which could alter certain prediction schemes, for further analyses.

### 2.2. A Subset of Wheat lncRNAs Contain Potential Precursors for miRNAs

LncRNAs and miRNAs are intricately linked; lncRNAs may harbor precursor sequences for miRNAs and can also be found in functional networks of target mimicry. Therefore, ‘clean’ putative lncRNA sequences were tested for the presence of miRNA precursors, using a two-step, homology-based approach [27]. In durum wheat CDC Fortitude, 136 putative lncRNA sequences contained precursors for 124 mature miRNAs belonging to 45 miRNA families. Similarly, 158, 139, 153, 156, and 137 putative lncRNAs from durum wheat varieties Kofa, Langdon, LDN-GB-3B, Pithless, and W9262 contained 128, 120, 120, 152, and 137 mature miRNA sequences from 40+ miRNA families, respectively (Supplementary Table S1). In order to provide support for predictions, all wheat miRNAs with small RNA (sRNA) sequencing evidence were extracted from the miRBase database (https://www.mirbase.org/, release 22.1;, accessed on 16 December 2022). Additional wheat miRNAs, not deposited to miRBase but still identified in the studies referencing these miRBase miRNAs, were included in a ‘validated’ set of 194 non-redundant miRNAs that combined sRNA evidence with precursor information, expression information from additional experimental techniques, and/or conservation information across plants. In addition to this ‘validated’ set, a second set of 2291 non-redundant miRNA sequences from recent sRNA sequencing studies, covering different stress conditions or developmental stages, were collected. This second set of ‘sRNA support’ miRNAs were primarily supported by only sRNA sequencing data. Unique mature miRNA sequences potentially carried on putative lncRNA sequences were compared to these sets of previously reported miRNA sequences for additional support (Supplementary Table S1). Of 124 lncRNA-derived unique miRNA sequences in CDC Fortitude, 27 were in the validated set and 35 were included in the sRNA support set. Together, 44 of the predicted unique miRNAs were reported previously, providing additional support for their authenticity. For Kofa, the same approach provided support for 55 of 128 unique miRNA sequences predicted from putative lncRNAs, of which 31 were in the validated set and 44 were in the sRNA support set. Following the same approach, additional evidence could be provided for the predicted miRNAs derived from lncRNAs as follows: 44 of the 120 (23 in validated set, 36 in sRNA support set) for Langdon; 40 of the 120 (23 in validated set, 32 in sRNA support set) for LDN-GB-3B; 57 of the 152 (31 in validated set, 44 in sRNA support set) for Pithless; and 50 of the 137 (27 in validated set, 41 in sRNA support set) for W9262-260D3 (Supplementary Table S1). It should be noted that miRNA expression is highly dynamic and often condition-, developmental stage- and environment-specific. Overall, precursor sequences for 68 miRNA families were found among all putative lncRNA transcripts from all durum varieties, where most families were shared across varieties, implying that miRNA-containing lncRNA transcripts may be relatively more conserved than the lncRNA sequences themselves.

In bread wheat varieties, 124 and 107 putative lncRNA transcripts from control 6218 and infested 6218 samples appeared to carry precursors for 127 and 102 mature miRNAs, respectively, representing 32 and 38 families. Control and infested samples from the resistant LX99 variety had 114 and 122 putative lncRNA sequences that harbored precursor sequences for 120 and 108 mature miRNAs from 38 and 40 families, respectively (Supplementary Table S2). Using a set of ‘validated’ and a set of ‘sRNA support’ miRNAs as detailed above, additional support was provided for the predicted unique miRNA sequences derived from lncRNAs as follows: 43 of the 127 (26 in validated set, 36 in sRNA support set) for non-infested 6128; 36 of the 102 (23 in validated set, 29 in sRNA support set) for infested 6218; 44 of the 120 (30 in validated set, 32 in sRNA support set) for non-infested LX99; and 35 of the 108 (25 in validated set, 27 in sRNA support set) for infested LX99. Mature miRNA sequences belonging to 58 families in total were identified across all putative lncRNA transcripts across all bread wheat samples, which may imply that stress conditions bring about diverse changes in the expression profiles of lncRNA-derived miRNAs.

Notably, a few lncRNA sequences appeared to carry potential precursor sequences for more than one miRNA family (Supplementary Tables S1 and S2). Among these, precursors for miRNA families such as [miR1120-miR1130/miR1122] and [miR1127-miR1135] were common to both durum and bread wheat samples. In durum wheat, precursor sequences for [miR1122-miR5175], [miR1127-miR1128], and [miR1117-miR1131] families were also predicted from putative lncRNA sequences from all varieties.

Prompted by apparent conservation at the miRNA family level, the mature miRNA sequences were compared across varieties and samples. Intriguingly, in contrast to lncRNA sequences themselves, predictions from both durum and bread wheat varieties suggested a high number of precursor sequences with the same mature miRNA sequences shared between varieties. For instance, 46 miRNAs with the same mature miRNA sequence were predicted from the putative lncRNA sequences from all durum wheat varieties (Supplementary Figure S2). Nevertheless, at the sequence level, many predicted miRNAs were still specific to single varieties and/or conditions (Supplementary Figure S2).

LncRNAs and miRNAs can be found in complex networks where a given lncRNA sequence may both harbor precursor sequences for miRNAs and contain target sites for miRNA-mediated regulation [28]. Therefore, we predicted target sites for miRNAs that are derived from lncRNA sequences among the putative lncRNA sequences. On average, ~600 lncRNA sequences were potentially targeted by one or more miRNAs predicted from the same sequences in durum wheat varieties. Similarly, ~660 lncRNA sequences from bread wheat samples were potentially targeted by lncRNA-derived miRNAs (Supplementary Tables S1 and S2). Among these, ~48 and 23 lncRNA sequences on average also contained predicted precursor sequences for miRNAs in durum and bread wheat samples, respectively. These figures suggest that roughly one-third of the putative lncRNAs that contained predicted precursors may also be involved in miRNA-mediated regulation networks in durum wheat. In bread wheat, such complex relationships existed for about one-fifth of predicted lncRNAs with predicted miRNA precursors. Among the putative lncRNAs that are both predicted sources and targets for miRNAs, there were instances where the same miRNA sequence could either be derived from the lncRNA and/or target the same lncRNA sequence (Supplementary Tables S1 and S2).

### 2.3. Genomic Loci Associated with Insect Resistance Contain Putative lncRNAs

Recently, we compared the genomic content and organization of important insect resistance loci in wheat and related cereals [20]. One of these loci, harboring the well-known 3B-QTL against Wheat Stem Sawfly (WSS) [21] and its immediate vicinity, had been mapped between 816,346,461 and 836,299,636 on the 3B chromosome of the *T. durum* Svevo genome (v1, GCA_900231445.1). Putative lncRNAs identified from durum wheat varieties were mapped to this location on the Svevo genome, identifying 148–211 putative lncRNAs from this locus (Supplementary Table S3). None of these lncRNAs contained any predicted precursors for miRNAs; however, one or two putative lncRNAs from each variety were potentially targeted by one or more miRNA families (Table 2). Intriguingly, miR1118 and miR1436 family members were predicted to target putative lncRNAs consistently, but only in the solid-stemmed CDC Fortitude, LDN-GB-3B, and W9262-260D3 varieties. LncRNAs from this locus were compared to the validated set of known wheat miRNAs, described earlier, to identify if these lncRNAs may also be modulated by miRNAs derived from elsewhere in the genome. Very few miRNAs from the validated set had the potential to target these lncRNAs, mostly in agreement with the lncRNA-derived miRNAs. Interestingly, however, two additional lncRNAs from solid-stemmed CDC Fortitude and W9262-260D3 varieties, not targeted by lncRNA-derived miRNAs, showed up as potential targets by the same miRNA (UCAGAUGAGAAGGCAGAUCAUA) of a yet-unknown family (Table 2). This miRNA shares moderate but not high sequence similarity to ata-miR9863b-5p and ata-miR9863b-3p from *Aegilops tauschii* (miRBase #MIMAT0037236 and #MIMAT0037105) at best.

Another insect resistance loci, around the well-known *Sm1* gene against OWBM, had been mapped to 10,885,794–38,440,107 on chromosome 2B on the *T. aestivum* Chinese Spring genome (IWGSC RefSeq v2.1) in our previous work [20]. Infested and control kernels from OWBM-susceptible and resistant bread wheat genotypes, 6218 and LX99, respectively, contained 97–145 putative lncRNAs at this locus (Supplementary Table S3). Similar to the observations from durum wheat varieties, these putative lncRNAs did not contain predicted precursor sequences for miRNAs. Putative lncRNAs from this locus in control kernels in the susceptible 6218 genotype did not appear to be targeted by miRNAs; however, kernels infested with OWBM from this genotype had two putative lncRNAs that are potentially targeted by miRNAs (Table 2). In the resistant LX99, putative lncRNAs in the control kernels had one sequence targeted by miR1127. In the infested LX99 kernels, one putative lncRNA was still predicted to be targeted by miR1127, in addition to another putative lncRNA potentially targeted by a second miRNA, miR5174 (Table 2). The validated set of known wheat miRNAs was also compared to the putative lncRNAs from the OWBM locus, which also suggested a few miRNAs from elsewhere in the wheat genome may target these lncRNAs, largely in agreement with lncRNA-derived miRNAs (Table 2). This analysis, however, identified one lncRNA from control kernels of the susceptible 6218 genotype that may be targeted by an miR9673 family member (UAAGAAGCAAAUAGCACAUG).

LncRNAs mapped to these insect resistance loci did not reveal any predicted targets in the *T. durum* Svevo and *T. aestivum* Chinese Spring transcriptomes. The miRNAs targeting these lncRNAs, however, did identify several predicted targets in these transcriptomes, which may indicate functional networks involving mRNAs–miRNAs–lncRNAs (Supplementary Table S3). Putative mRNA targets for both durum and bread wheat samples had diverse assumed functions. Among these, interestingly, the miR1118 family members potentially targeting putative lncRNAs in all solid-stemmed durum wheat varieties also potentially targeted an NB-ARC domain-containing protein. The miR1436 family, similarly targeting putative lncRNAs in all solid-stemmed varieties, was predicted to regulate multiple targets in all three varieties, which included stress-related genes. Beyond the miRNAs predicted from putative lncRNAs, previously known wheat miRNAs from the validated miRNA set, as detailed earlier, also suggested mRNA–miRNA–lncRNA networks; a few known wheat miRNAs appeared to target the same lncRNAs and mRNAs in the same genotype, as well as a few others, which suggested additional mRNA targets (Supplementary Table S3). Among the latter, a previously reported wheat miRNA with no significant similarities to known miRNA families was predicted to target two lncRNAs from the solid-stemmed CDC Fortitude and LDN-GB-3B (Table 2), along with several predicted mRNA targets that included stress-related genes (Supplementary Table S3). In bread wheat samples, miR1128, predicted to target STRG.26323.1 only in infested kernels of OWBM-susceptible 6218, was also predicted to target an NB-ARC domain-containing protein. None of the predicted target transcripts for the miR1127 family were specific to infested kernels of the resistant LX99 variety. On the other hand, the miR5174 family, predicted to target STRG.31924.1 only in infested LX99 kernels, did not have predicted target transcripts with an apparent function in disease resistance (Table 2, Supplementary Table S3). Known wheat miRNAs from the validated set largely had the same putative lncRNAs and mRNAs as potential targets in the same genotypes. These known miRNAs, which may come elsewhere in the genome, suggested only a few additional predicted targets, almost all of which are currently uncharacterized (Supplementary Table S3).

### 2.4. Some lncRNAs May Derive from Organellar Genomes

In plants, whether or not chloroplast and mitochondrial genomes contribute to the ncRNA pool has not been firmly established yet, even though recent observations suggest this may be possible. We compared putative clean lncRNA sequences from bread wheat genotypes to *T. aestivum* reference chloroplast (NCBI Refseq: NC_002762.1) and mitochondrial (NCBI Refseq: NC_036024.1) genomes. Similarly, putative clean lncRNAs from durum wheat varieties were compared to *T. durum* chloroplast (GenBank: MZ230674.1) and mitochondrial (GenBank: KJ078649.1) genomes deposited in NCBI. At and above 98% sequence identity across at least 98% of the lncRNA length, several lncRNA sequences from all samples matched both organellar genomes, suggesting that these may indicate lncRNA sequences of non-nuclear origin (Supplementary Table S4). Intriguingly, even though the total number of putative lncRNAs was much higher in bread wheat samples, the number of putative lncRNAs that match organellar genomes was higher in durum wheat genotypes. Overall, more lncRNAs mapped to the mitochondrial genomes than chloroplast genomes, which roughly match the size differences of two organellar genomes. None of the lncRNAs that significantly matched organellar genomes contained any potential precursors for miRNAs. Albeit comparatively few, these observations suggest that organellar genomes may contribute to the lncRNA pool of the cells.

## 3. Discussion

Our current understanding of how genomes work suggests that up to 90% of our genomes are transcribed throughout our life, some at different developmental stages, or others during some specific conditions. However, only a small portion of these transcribed sequences correspond to protein coding genes. It is not yet clear if this transcriptional activity is “transcriptional noise” in large part, or, if not, the functional implications are not well-understood. Yet, recent observations from flowering plants suggest about 40% of the “noncoding” transcripts are likely functional [1,29]. Various studies demonstrating the involvement of noncoding transcripts under diverse environments/conditions support this view [26].

In this study, we explored long noncoding RNAs (lncRNAs) among assembled transcripts from durum and bread wheat varieties. For durum wheat, RNA-Sequencing (RNA-Seq) data from stem tissues of solid-stemmed CDC Fortitude, LDN-GB-3B, W9262-2603D, and hollow-stemmed Kofa, Langdon, and Pithless varieties were first assembled into transcripts. For bread wheat, RNA-Seq data, used for transcriptome assembly, originated from kernels with or without infestation with Orange Wheat Blossom Midge (OWBM), from LX99 and 6218 varieties, resistant and susceptible to this pest, respectively. By progressively eliminating transcripts matching other types of noncoding RNAs and transcripts with long ORFs, coding potentials, or extensive homology to coding sequences, we identified 33–38 k putative lncRNAs in tetraploid durum wheat varieties and 42–57k lncRNAs in hexaploid bread wheat varieties. Overall, putative lncRNA contents, identified from untreated stem transcriptomes, were similar across durum wheat varieties. The durum wheat varieties included in this study differ in their stem structure, which plays a major role in defense against the devastating pest, Wheat Stem Sawfly (WSS). Accordingly, the transcriptome data were derived from a developmental stage where stem pith breakdown begins in hollow-stemmed varieties [21]. Therefore, it could be argued that, besides stem structure changes, these plants were, overall, in a similar metabolic state, reflected in the overall contents of lncRNAs, as well. In contrast, putative lncRNA contents between bread wheat samples appeared to vary widely (Table 1). Notably, the resistant LX99 variety had an overall higher content of lncRNAs, which further increased upon infestation. However, lncRNA content appeared to decrease in response to OWBM infestation in the susceptible 6218 variety. It is tempting to speculate that response to OWBM infestation may involve action of lncRNAs, in addition to causal genes, such as *Sm1* [22]. Nevertheless, in terms of lncRNA identities, different varieties and/or conditions (control vs. infested) are characterized by high numbers of genotype- or condition-specific lncRNAs (Figure 1 and Figure 2). Taken together, similar total lncRNA contents seem to not necessarily indicate similar lncRNA profiles at the sequence level; however, lncRNAs divergent at the sequence level may still have functional overlaps.

The involvement of lncRNA as regulatory molecules within several aspects of plant growth and development, including stress responses, has been well established [18,26]. Recent studies also uncovered functional links between lncRNAs and microRNAs (miRNAs), another type of regulatory, noncoding small RNAs [3,30]. Some lncRNA transcripts have been shown to contain precursor sequences for these small regulatory molecules and/or to be targeted by them. In line with these findings, a small portion of the putative lncRNA transcripts identified from durum and bread wheat varieties contained predicted precursor sequences for diverse miRNA families (Supplementary Tables S1 and S2). Predicted mature miRNA sequences were compared to two sets of previously reported wheat miRNAs: (1) a ‘validated’ set from the miRBase database with small RNA-sequencing (sRNA-seq) data, in addition to other evidence, including precursor sequences, expression by independent techniques, and conservation across plants, and (2) an ‘sRNA-seq support’ set that is composed of previously reported wheat miRNAs with primarily sRNA-seq evidence. This comparison indicated that 32–43% of predicted mature miRNA sequences had been previously reported in wheat, thereby providing support to their authenticity. Considering that miRNAs often have highly dynamic and condition-specific expression profiles, a lack of such a support does not necessarily deny authenticity. The predicted miRNA families derived from lncRNA sequences included a few well-characterized, highly conserved families that have been previously reported in association with lncRNAs [28,31]. For instance, lncRNAs compete with the endogenous target of the highly conserved, stress-responsive miR398, modulating the cold response in winter wheat [32]. In Kofa, putative lncRNAs STRG.38906.1 and STRG.38906.2 had potential precursor sequences for miR398, which was predicted to target another lncRNA, STRG.61775.1. Other conserved families, miR160 and miR166, have also been validated as endogenous target mimics in *Arabidopsis* [31]. Putative lncRNA sequences from all but one sample (6218 infested) contained predicted precursor sequences for both miR160 and miR166 families, in addition to being potentially targeted by these families. In another recent study, the miR166 family, in addition to miR156, and miR172 family members, had been found in complex networks with lncRNAs, where they could either be processed from lncRNAs, target lncRNAs, or modulated through target mimicry by lncRNAs [28]. Similarly, precursors within lncRNA sequences were found for miR156, miR169, and miR394 families, which were implicated in pathogen responses [33,34].

Additionally, a few putative lncRNAs contained predicted precursors for multiple miRNA families. Besides a few well-known families, these included mostly miRNA families that are poorly characterized at best. Therefore, whether the presence of multiple precursors within lncRNAs indicates a multi-layered control over specific pathways will await functional characterization of the specific families in question. However, in light of a recent study that suggests miR1120 and miR1122 families work together in anther development in wheat [35], multiple precursor sequences within single lncRNAs may indeed represent functional networks. Precursor sequences for both of these families were found on putative lncRNAs from all varieties/samples in our study (Supplementary Tables S1 and S2).

In our previous work, we compared the content and organization of genomic loci associated with responses against two important wheat pests, WSS and OWBM, in wheat and related cereals [20]. At these genomic intervals, we identified 148–211 putative lncRNAs for durum wheat varieties and 97–145 putative lncRNAs for bread wheat samples (Table 2). These putative lncRNA sequences did not contain precursors for miRNAs; however, very few were predicted to be targeted by miRNAs, derived from other lncRNA sequences. Notably, miR1118, miR1436, miR1130, and miR5174 families had predicted targets only in the solid-stemmed varieties. Of these, miR1436 has been implicated in stress responses before [36]. Additionally, while a direct link was not established between miR1436 and biosynthesis of terpenes, secondary metabolites that protect plants against herbivorous attacks, this family was among the top predictions in *Ferula gummosa*, a plant that is used for medicinal and industrial purposes related to terpenes. In that work, two other families, miR5658 and miR5021, also from the top predictions, were linked to terpene biosynthesis [37]. Accordingly, predicted targets of miR1436 included genes associated with stress responses (VAH71766.1, VAH56251.1, VAI58926.1) and genome structure and maintenance (VAH16302.1, VAI27812.1, VAH31495.1) (Supplementary Table S3). While the locus on chromosome 3B in durum wheats primarily affects stem structure, which provides a physical defense against WSS [21], coding and noncoding features at and around this genomic loci can still contribute to WSS resistance through additional mechanisms. Along with chromatin modification, miR1127-mediated regulation of WRKY75 transcription factor was implicated in stress responses in a recent study [38]. miR1127 was predicted to target two different lncRNAs in the infested kernels of OWBM-resistant LX99 genotype (Table 2). It is tempting to speculate whether miR1127 acts as a master regulator of biotic stress response in this genotype. As suggested by the predicted targets of lncRNA-derived miRNAs that potentially target other lncRNAs from stress-related loci, stress responses may govern players of core biological pathways, such as replication, transcription, and translation, in addition to well-known stress-resistance genes.

In addition to lncRNA-derived miRNAs, lncRNAs from WSS- and OWBM-related genomic loci can be regulated by miRNAs derived from elsewhere in the genome. Therefore, we used the validated wheat miRNA set to look for predicted targets among the lncRNAs, as well as transcripts, from these genomic loci. Target predictions with the validated set matched predictions with the lncRNA-derived miRNAs in most cases (Supplementary Table S3). However, particularly in durum wheat, a previously reported miRNA with a yet-unknown family suggested links between lncRNAs from solid-stemmed CDC Fortitude and LDN-GB-3B and many more transcripts, which included those that belong to stress-related families (Table 2, Supplementary Table S3). It is therefore tempting to speculate whether this locus, primarily controlling stem structure, may still contribute to WSS response through additional mechanisms that involve noncoding transcripts.

A newly emerging function for lncRNAs in non-plant systems involves intracellular signaling between the nucleus and the endosymbiotic organelles, chloroplast and mitochondria [39]. While it is not yet fully clear whether lncRNAs are processed from these genomes, particularly chloroplasts, recent observations suggest they may be [40]. In our work, we observed putative lncRNAs that match both chloroplast and mitochondrial genomes at high sequence identity and coverage of the lncRNA (Supplementary Table S4). We did not observe any potential precursor sequences within these lncRNAs, however, organellar genomes do appear to contribute to the lncRNA pool of the cell, roughly proportional to their sizes, in the developmental stages and/or treatments of the wheat samples used in this study. Given the major roles of chloroplast and mitochondria in energy metabolism, it would not be surprising if these organelles are involved in the ncRNA-mediated regulation of gene expression.

## 4. Materials and Methods

### 4.1. RNA-Sequencing Data and Transcriptome Assembly

RNA-Sequencing (RNA-Seq) data from durum wheat lines CDC Fortitude, Kofa, Langdon, LDN-GB-3B, W9262-260D3, and Pithless (SRR12041536-SRR12041553) were downloaded from NCBI (https://www.ncbi.nlm.nih.gov/bioproject/PRJNA630287, accessed on 16 December 2022) [21] Similarly, RNA-Seq data from bread wheat lines LX99 and 6218 (SRR16962361-SRR16962372) were downloaded from NCBI (https://www.ncbi.nlm.nih.gov/bioproject/PRJNA780663, accessed on 16 December 2022) [25]. Adapter sequences and low-quality bases were removed using Trimmomatic v38 (ILLUMINACLIP:TruSeq3-PE-2.fa:2:30:10 LEADING:3 TRAILING:3 MAXINFO:40:0.8 MINLEN:36) [41]. Clean reads from durum wheat varieties were mapped on the *Triticum turgidum* L. ssp. *durum* Svevo genome (v1, GenBank assembly accession: GCA_900231445.1) [42] using hisat2 using default parameters [43]. Clean reads from bread wheat genotypes were also mapped with hisat2 using default parameters, on the *Triticum aestivum* Chinese Spring genome (IWGSC RefSeq v2.1, wheat-urgi.versailles.inra.fr) [44]. SAM files generated by hisat2 were converted into BAM files using *samtools view*, and replicate BAM files were combined with *samtools merge* and sorted using *samtools sort* (version 1.15, http://www.htslib.org/doc/samtools.html, accessed on 16 December 2022) [45]. Finally, transcript sequences were extracted using StringTie (stringtie2.2.1, -m 100 -s 1) [46]. Fasta sequences from StringTie GTF output were obtained using gffread v0.12.7 (https://github.com/gpertea/gffread) [47]. These assembled sequences will be referred to as ‘transcriptomes’ or ‘transcripts’ hereafter for simplicity.

### 4.2. In Silico Identification of Long Noncoding RNAs

Putative lncRNAs were identified using a tiered procedure, based on slight modifications of widely accepted criteria [4,10], as detailed below. In the first step, all known *Triticum* tRNA (8290 sequences), snoRNA (5313 sequences), snRNA (2123 sequences), and rRNA (9461 sequences) were retrieved from RNAcentral v20 (https://rnacentral.org/). Unrelated sequences coming from batch search and download were removed using custom python3 scripts, leaving a total of 23,864 known *Triticum* RNA sequences. These sequences were blasted against the transcriptomes from durum and bread wheat varieties as described above, using standalone BLAST 2.11.0+ (blastn tool, e-value 10-10) [48]. Transcripts matching a known RNA by more than 10% of its length (query coverage) or covering more than 10% of a known RNA (subject coverage) were discarded, along with the transcripts that were shorter than 200 nucleotides. In the second step, potential ORF sizes in the transcript sequences were calculated using Transdecoder v5.5.0 (https://github.com/TransDecoder/TransDecoder). From this step, all transcripts containing potential ORFs larger than 100 amino acids were discarded. In the third step, coding potentials of the remaining candidates were predicted using CPC 2.0 (http://cpc2.gao-lab.org/) [49] and CPAT v3.0.4 (https://cpat.readthedocs.io/en/latest/#) [50]. For CPAT predictions for the durum wheat varieties, a custom hexamer table and logit model were constructed by training CPAT with 190,470 coding sequences from Svevo reference annotation v1 (GCA_900231445) and a combination of 23,864 clean known *Triticum* RNAs described above and 12,427 wheat noncoding sequences from NONCODE v6 database (http://www.noncode.org/, accessed on 16 December 2022). Note that NONCODE v6 database sequences contain lncRNA sequences, which were, thus, not used in the first filtering step. The same noncoding sequences were used, along with 126,244 high-confidence coding sequences from *T. aestivum* Chinese Spring reference annotation v2.1 (IWGSC RefSeq v2.1), to train CPAT prior to making predictions for the bread wheat varieties. CPAT noncoding predictions below a probability cutoff of 0.00001 were accepted. Transcript sequences that were deemed as noncoding by both CPC2 and CPAT were retained. In the final step, homology searches were performed between candidate lncRNAs and coding sequences. All remaining transcripts were compared against all Svevo reference v1 (GCA_900231445) and Chinese Spring reference v2.1 (IWGSC RefSeq v2.1) coding sequences using blastn (e-value 10-10 ). Transcript sequences matching a coding sequence by more than 20% of their lengths (query coverage) were removed. Transcript sequences yielding no matches to coding sequences were accepted as ‘clean’ putative lncRNAs. Transcript sequences with matching to coding sequences up to 20% of their lengths were considered as putative lncRNAs ‘with partial matches’ to coding sequences.

Clustering of putative lncRNA sequences was done with CD-HIT at 90% sequence identity (cd-hit-est, -c 0.9 -n 8) [51]. Each cluster generated by CD-HIT potentially corresponds to the same putative lncRNA in different varieties. Clusters shared by different varieties were visualized by UpSetR (https://vdl.sci.utah.edu/publications/2017_bioinformatics_upsetr/) [52].

LncRNAs mapping to the insect-resistant loci delineated in our previous work [20] were identified using a strict mapping approach. In the first step, putative lncRNAs were mapped to the Svevo chromosome 3B (816,346,461 to 836,299,636) for durum wheat samples and to Chinese Spring chromosome 2B (10,885,794 to 38,440,107) for bread wheat samples using blastn (e-value 10-10 ). Significant hits with 100% coverage of the lncRNA sequence and >95% identity were kept. Then, these lncRNAs were mapped to the respective genomes (blastn, e-value 10-10 -max_hsps 10 -perc_identity 95 -qcov_hsp_perc 0.8). LncRNAs that mapped to another location with a higher percent identity were discarded.

### 4.3. In Silico Identification of microRNAs and Prediction of Targets

Putative ‘clean’ lncRNA sequences were analyzed for the presence of miRNA precursors, using a fully automated pipeline that employs a homology-based approach [27]. Briefly, mature miRNA sequences for *Viridiplantae* species were retrieved from the miRBase database (Release 22.1, https://www.mirbase.org/, accessed on 16 December 2022) and compared to the lncRNA sequences, allowing, at most, 1 mismatch. For significant matches, precursor sequences were extracted, folded, and assessed for known pre-miRNA characteristics: (1) no mismatches at Dicer cut sites, (2) no multi-branched loops, (3) no overlaps between the mature miRNA sequence and the head portion of the hairpin, (4) no more than four and six mismatches between miRNA and miRNA* [27].

Potential targets of miRNAs were identified using psRNATarget web server, with Expectation = 3 and maximum UPE = 25 (https://www.zhaolab.org/psRNATarget/analysis, accessed on 16 December 2022) [53]. Probable functions of target transcripts are deduced through their homology with annotated proteins. To do so, UniRef100 (clusters of protein sequences at 100% identity), filtered by taxonomy Triticum [4564] (406,133 sequences), was downloaded from the UniProt database (https://www.uniprot.org/, accessed on 16 December 2022). Predicted targets were compared to these protein sequences using blastx (e-value 10-6 ). Significant hits were filtered for a minimum 50% identity and minimum 75% coverage of the transcript. Best hits were selected based on % identity and transcript coverage next.

To provide support to miRNA predictions and expand the target analyses, two sets of known wheat miRNAs were generated: (1) A ‘validated’ set of known wheat miRNAs that included all wheat miRNAs deposited in the miRBase (Release 22.1), referenced by small RNA sequencing studies [54,55,56], in addition to miRNAs reported in these studies but not deposited. These miRNAs largely included additional evidence, including valid precursor sequences, expression data by independent techniques, and conservation with other plants. (2) An ‘sRNA-seq support’ set of wheat miRNAs that included a diverse set of miRNAs reported in recent small RNA sequencing studies in wheat that covered different stress conditions and/or developmental stages [57,58,59,60,61,62,63]. In this set, the primary evidence comes from small RNA sequencing data alone.

### 4.4. Comparison of the Putative lncRNAs to the Organellar Genomes

*T. aestivum* reference chloroplast (NCBI Refseq: NC_002762.1) and mitochondrial (NCBI Refseq: NC_036024.1) genomes were retrieved from NCBI. Reference sequences for *T. durum* chloroplast and mitochondria were not available; therefore, complete chloroplast (GenBank: MZ230674.1) and mitochondrial (GenBank: KJ078649.1) genomes were retrieved from NCBI for this species. Putative lncRNAs were compared to these genomes using blastn (-evalue 10-10 ). Hits with at least 98% coverage of the lncRNA sequence and at least 98% identity were deemed significant. It should be noted that the *T. durum* mitochondrial genome is listed as ‘unverified’ in NCBI as of October 2022, even though this sequence was reported in a peer-reviewed study [64]. To ensure the reliability of the data, durum wheat lncRNAs were compared to both *T. durum* and *T. aestivum* mitochondrial genomes. Significant hits of at least 98% sequence identity and lncRNA coverage were the same for both mitochondrial genomes across all genotypes, except for W9262-260D3, where the *T. aestivum* mitochondrial genome yielded one additional significant hit. Therefore, results with the durum mitochondrial genome (Genbank: KJ078649.1) for durum wheat genotypes are presented in this study.

All other analyses of combining/comparing datasets, as well as blastn/blastx filtering, were done with custom Python3 scripts.

## Figures and Tables

**Table 1:** The total number of assembled transcripts and transcripts passing each tier in the lncRNA identification procedure in durum and bread wheat varieties.

Variety/ Sample	Total Number of Assembled Transcripts	Number of Transcripts Remaining after Removal of Other Noncoding RNA Types (>200 nt)	Number of Transcripts with Potential ORFs < 100 aa	Number of Transcripts with No Coding Potential—CPAT	Number of Transcripts with No Coding Potential—CPC2	Number of Transcripts with No Coding Potential—BOTH (Hence Retained)	Number of Transcripts Not Matching Coding Sequences	Number of Transcripts Partially Matching Coding Sequences
*Triticum durum* (AABB) varieties								
**CDC Fortitude**	173,462	168,753	57,278	55,589	54,847	53,519	38,356	6075
**LDN-GB-3B**	161,633	157,632	51,472	49,875	49,351	48,063	34,122	5443
**W9262-260D3**	160,015	155,787	51,128	49,523	48,932	47,673	33,832	5646
**Kofa**	170,654	166,414	55,698	53,985	53,398	52,032	37,210	5897
**Langdon**	162,270	158,438	51,900	50,241	49,553	48,263	33,946	5796
**Pithless**	165,947	161,925	53,462	51,825	51,259	49,955	35,386	5722
*Triticum aestivum* (AABBDD) varieties								
**6218 Control**	214,161	209,624	72,672	69,717	69,387	67,398	47,146	5874
**6218 Infested**	202,040	197,970	66,991	64,270	64,118	62,225	42,427	5461
**LX99 Control**	210,529	206,501	77,489	74,829	74,596	72,724	51,560	5188
**LX99 Infested**	219,575	215,276	85,389	82,452	82,164	80,156	57,166	5640

**Table 2:** Putative lncRNAs from durum and bread wheat varieties, mapping to two important insect resistance loci on 3B and 2B chromosomes, against WSS and OWBM, respectively, and lncRNA-derived miRNAs and known wheat miRNAs potentially targeting these lncRNAs.

Variety/ Sample	No. of lncRNAs from Insect Resistance Loci	LncRNAs from Insect Resistance Potentially Targeted by miRNAs		Targeting miRNA Families Predicted from lncRNAs	Targeting Known Wheat miRNA Families
*Triticum durum (AABB) varieties*					
**CDC Fortitude**	177		STRG.49702.1	miR1118, miR1436, miR5174	miR1118
			STRG.49256.1	-	Unknown family
**LDN-GB-3B**	164		STRG.46147.1	miR1118, miR1436	miR1118
			STRG.46190.1	miR1130_miR1122	miR1130
			STRG.45094.1	-	Unknown family
**W9262-260D3**	148		STRG.46057.1	miR1118, miR1130, miR1436, miR5174,	miR1118, miR1138
			STRG.46179.1	miR5174, miR5181	-
**Kofa**	211		STRG.49467.1	miR395	-
**Langdon**	180		STRG.49026.1	miR169	-
			STRG.45799.1	miR169	miR6197
**Pithless**	159		STRG.47553.1	miR1439	-
*Triticum aestivum (AABBDD) varieties*					
**6218 Control**	109		STRG.29249.1	-	miR9673
**6218 Infested**	97		STRG.26323.1	miR1128	-
			STRG.26415.1	mR6197	-
**LX99 Control**	108		STRG.30297.1	miR1127	miR1127
**LX99 Infested**	145		STRG.31964.1	miR1127	miR1127
			STRG.31924.1	miR5174	miR1133

## Data Availability

Not applicable.

## Supplementary Materials

The following supporting information can be downloaded at: https://www.mdpi.com/article/10.3390/ijms24032226/s1.

## References

[1] Kung J.T.Y., Colognori D., Lee J.T. (2013). Long Noncoding RNAs: Past, Present, and Future. Genetics.

[2] Deng P., Liu S., Nie X., Weining S., Wu L. (2017). Conservation Analysis of Long Non-Coding RNAs in Plants. Sci. China Life Sci..

[3] Brant E.J., Budak H. (2018). Plant Small Non-Coding RNAs and Their Roles in Biotic Stresses. Front. Plant Sci..

[4] Budak H., Kaya S.B., Cagirici H.B. (2020). Long Non-Coding RNA in Plants in the Era of Reference Sequences. Front. Plant Sci..

[5] Núñez-Martínez H.N., Recillas-Targa F. (2022). Emerging Functions of LncRNA Loci beyond the Transcript Itself. Int. J. Mol. Sci..

[6] Wierzbicki A.T., Blevins T., Swiezewski S. (2021). Long Noncoding RNAs in Plants. Annu. Rev. Plant Biol..

[7] Zhao Z., Zang S., Zou W., Pan Y.B., Yao W., You C., Que Y. (2022). Long Non-Coding RNAs: New Players in Plants. Int. J. Mol. Sci..

[8] Holley C.L., Topkara V.K. (2011). An Introduction to Small Non-Coding RNAs: MiRNA and SnoRNA. Cardiovasc. Drugs Ther..

[9] Hung T., Chang H.Y. (2011). Long Noncoding RNA in Genome Regulation: Prospects and Mechanisms. RNA Biol..

[10] Cagirici H.B., Biyiklioglu S., Budak H. (2017). Assembly and Annotation of Transcriptome Provided Evidence of MiRNA Mobility between Wheat and Wheat Stem Sawfly. Front. Plant Sci..

[11] Cech T.R., Steitz J.A. (2014). The Noncoding RNA Revolution—Trashing Old Rules to Forge New Ones. Cell.

[12] Jin J., Lu P., Xu Y., Li Z., Yu S., Liu J., Wang H., Chua N.H., Cao P. (2021). PLncDB V2.0: A Comprehensive Encyclopedia of Plant Long Noncoding RNAs. Nucleic Acids Res..

[13] Zhao L., Wang J., Li Y., Song T., Wu Y., Fang S., Bu D., Li H., Sun L., Pei D. (2021). NONCODEV6: An Updated Database Dedicated to Long Non-Coding RNA Annotation in Both Animals and Plants. Nucleic Acids Res..

[14] Sharma S., Taneja M., Tyagi S., Singh K., Upadhyay S.K. (2017). Survey of High Throughput RNA-Seq Data Reveals Potential Roles for LncRNAs during Development and Stress Response in Bread Wheat. Front. Plant Sci..

[15] Chen Q., Liu K., Yu R., Zhou B., Huang P., Cao Z., Zhou Y., Wang J. (2021). From “Dark Matter” to “Star”: Insight Into the Regulation Mechanisms of Plant Functional Long Non-Coding RNAs. Front. Plant Sci..

[16] Chen J., Zhong Y., Qi X. (2021). LncRNA TCONS_00021861 Is Functionally Associated with Drought Tolerance in Rice (Oryza Sativa L.) via Competing Endogenous RNA Regulation. BMC Plant Biol..

[17] Li C., Nong W., Zhao S., Lin X., Xie Y., Cheung M.Y., Xiao Z., Wong A.Y.P., Chan T.F., Hui J.H.L. (2022). Differential MicroRNA Expression, MicroRNA Arm Switching, and MicroRNA:Long Noncoding RNA Interaction in Response to Salinity Stress in Soybean. BMC Genom..

[18] Zhang H., Guo H., Hu W., Ji W. (2020). The Emerging Role of Long Non-Coding RNAs in Plant Defense against Fungal Stress. Int. J. Mol. Sci..

[19] Biyiklioglu S., Alptekin B., Akpinar B.A., Varella A.C., Hofland M.L., Weaver D.K., Bothner B., Budak H. (2018). A Large-Scale Multiomics Analysis of Wheat Stem Solidness and the Wheat Stem Sawfly Feeding Response, and Syntenic Associations in Barley, Brachypodium, and Rice. Funct. Integr. Genom..

[20] Muslu T., Akpinar B.A., Biyiklioglu-Kaya S., Yuce M., Budak H. (2021). Comparative Analysis of Coding and Non-Coding Features within Insect Tolerance Loci in Wheat with Their Homologs in Cereal Genomes. Int. J. Mol. Sci..

[21] Nilsen K.T., Walkowiak S., Xiang D., Gao P., Quilichini T.D., Willick I.R., Byrns B., N’Diaye A., Ens J., Wiebe K. (2020). Copy Number Variation of TdDof Controls Solid-Stemmed Architecture in Wheat. Proc. Natl. Acad. Sci. USA.

[22] Walkowiak S., Gao L., Monat C., Haberer G., Kassa M.T., Brinton J., Ramirez-Gonzalez R.H., Kolodziej M.C., Delorean E., Thambugala D. (2020). Multiple Wheat Genomes Reveal Global Variation in Modern Breeding. Nature.

[23] Ma K., Shi W., Xu M., Liu J., Zhang F. (2018). Genome-Wide Identification and Characterization of Long Non-Coding RNA in Wheat Roots in Response to Ca2+ Channel Blocker. Front. Plant Sci..

[24] Madhawan A., Sharma A., Bhandawat A., Rahim M.S., Kumar P., Mishra A., Parveen A., Sharma H., Verma S.K., Roy J. (2020). Identification and Characterization of Long Non-Coding RNAs Regulating Resistant Starch Biosynthesis in Bread Wheat (Triticum Aestivum L.). Genomics.

[25] Wang Q., Liu X., Liu H., Fu Y., Cheng Y., Zhang L., Shi W., Zhang Y., Chen J. (2022). Transcriptomic and Metabolomic Analysis of Wheat Kernels in Response to the Feeding of Orange Wheat Blossom Midges (Sitodiplosis Mosellana) in the Field. J. Agric. Food Chem..

[26] Jha U.C., Nayyar H., Jha R., Khurshid M., Zhou M., Mantri N., Siddique K.H.M. (2020). Long Non-Coding RNAs: Emerging Players Regulating Plant Abiotic Stress Response and Adaptation. BMC Plant Biol..

[27] Cagirici H.B., Sen T.Z., Budak H. (2021). Mirmachine: A One-Stop Shop for Plant Mirna Annotation. J. Vis. Exp..

[28] Ke L., Zhou Z., Xu X.W., Wang X., Liu Y., Xu Y., Huang Y., Wang S., Deng X., Chen L.L. (2019). Evolutionary Dynamics of LincRNA Transcription in Nine Citrus Species. Plant J..

[29] Yu Y., Chen X., Chen Y. (2019). Plant Noncoding RNAs: Hidden Players in Development and Stress Responses. Annu. Rev. Cell Dev. Biol..

[30] Budak H., Akpinar A. (2011). Dehydration Stress-Responsive Mirna in Brachypodium Distachyon: Evident by Genome-Wide Screening of Micrornas Expression. Omi. A J. Integr. Biol..

[31] Wu H.J., Wang Z.M., Wang M., Wang X.J. (2013). Widespread Long Noncoding RNAs as Endogenous Target Mimics for MicroRNAs in Plants. Plant Physiol..

[32] Lu Q., Guo F., Xu Q., Cang J. (2020). LncRNA Improves Cold Resistance of Winter Wheat by Interacting with MiR398. Funct. Plant Biol..

[33] Joshi R.K., Megha S., Basu U., Rahman M.H., Kav N.N.V. (2016). Genome Wide Identification and Functional Prediction of Long Non-Coding RNAs Responsive to Sclerotinia Sclerotiorum Infection in Brassica Napus. PLoS ONE.

[34] Zhang Y.Y., Hong Y.H., Liu Y.R., Cui J., Luan Y.S. (2021). Function Identification of MiR394 in Tomato Resistance to Phytophthora Infestans. Plant Cell Rep..

[35] Sun L., Sun G., Shi C., Sun D. (2018). Transcriptome Analysis Reveals New MicroRNAs-Mediated Pathway Involved in Anther Development in Male Sterile Wheat. BMC Genom..

[36] Mangrauthia S.K., Bhogireddy S., Agarwal S., Prasanth V.V., Voleti S.R., Neelamraju S., Subrahmanyam D. (2017). Genome-Wide Changes in MicroRNA Expression during Short and Prolonged Heat Stress and Recovery in Contrasting Rice Cultivars. J. Exp. Bot..

[37] Sobhani Najafabadi A., Naghavi M.R. (2018). Mining Ferula Gummosa Transcriptome to Identify MiRNAs Involved in the Regulation and Biosynthesis of Terpenes. Gene.

[38] López-Galiano M.J., González-Hernández A.I., Crespo-Salvador O., Rausell C., Real M.D., Escamilla M., Camañes G., García-Agustín P., González-Bosch C., García-Robles I. (2018). Epigenetic Regulation of the Expression of WRKY75 Transcription Factor in Response to Biotic and Abiotic Stresses in Solanaceae Plants. Plant Cell Rep..

[39] Calderon R.H., Strand Å. (2021). How Retrograde Signaling Is Intertwined with the Evolution of Photosynthetic Eukaryotes. Curr. Opin. Plant Biol..

[40] Zhelyazkova P., Sharma C.M., Forstner K.U., Liere K., Vogel J., Borner T. (2012). The Primary Transcriptome of Barley Chloroplasts: Numerous Noncoding RNAs and the Dominating Role of the Plastid-Encoded RNA Polymerase. Plant Cell.

[41] Bolger A.M., Lohse M., Usadel B. (2014). Trimmomatic: A Flexible Trimmer for Illumina Sequence Data. Bioinformatics.

[42] Maccaferri M., Harris N.S., Twardziok S.O., Pasam R.K., Gundlach H., Spannagl M., Ormanbekova D., Lux T., Prade V.M., Milner S.G. (2019). Durum Wheat Genome Highlights Past Domestication Signatures and Future Improvement Targets. Nat. Genet..

[43] Kim D., Paggi J.M., Park C., Bennett C., Salzberg S.L. (2019). Graph-Based Genome Alignment and Genotyping with HISAT2 and HISAT-Genotype. Nat. Biotechnol..

[44] International Wheat Genome Sequencing Consortium (2018). Shifting the Limits in Wheat Research and Breeding Using a Fully Annotated Reference Genome. Science.

[45] Danecek P., Bonfield J.K., Liddle J., Marshall J., Ohan V., Pollard M.O., Whitwham A., Keane T., McCarthy S.A., Davies R.M. (2021). Twelve Years of SAMtools and BCFtools. Gigascience.

[46] Pertea M., Pertea G.M., Antonescu C.M., Chang T.C., Mendell J.T., Salzberg S.L. (2015). StringTie Enables Improved Reconstruction of a Transcriptome from RNA-Seq Reads. Nat. Biotechnol..

[47] Pertea G., Pertea M. (2020). GFF Utilities: GffRead and GffCompare. F1000Research.

[48] Camacho C., Coulouris G., Avagyan V., Ma N., Papadopoulos J., Bealer K., Madden T.L. (2009). BLAST+: Architecture and Applications. BMC Bioinform..

[49] Kang Y.J., Yang D.C., Kong L., Hou M., Meng Y.Q., Wei L., Gao G. (2017). CPC2: A Fast and Accurate Coding Potential Calculator Based on Sequence Intrinsic Features. Nucleic Acids Res..

[50] Wang L., Park H.J., Dasari S., Wang S., Kocher J.P., Li W. (2013). CPAT: Coding-Potential Assessment Tool Using an Alignment-Free Logistic Regression Model. Nucleic Acids Res..

[51] Li W., Godzik A. (2006). Cd-Hit: A Fast Program for Clustering and Comparing Large Sets of Protein or Nucleotide Sequences. Bioinformatics.

[52] Conway J.R., Lex A., Gehlenborg N. (2017). UpSetR: An R Package for the Visualization of Intersecting Sets and Their Properties. Bioinformatics.

[53] Dai X., Zhuang Z., Zhao P.X. (2018). PsRNATarget: A Plant Small RNA Target Analysis Server (2017 Release). Nucleic Acids Res..

[54] Yao Y., Guo G., Ni Z., Sunkar R., Du J., Zhu J.K., Sun Q. (2007). Cloning and Characterization of MicroRNAs from Wheat (Triticum Aestivum L.). Genome Biol..

[55] Han R., Jian C., Lv J., Yan Y., Chi Q., Li Z., Wang Q., Zhang J. (2014). Identification and Characterization of MicroRNAs in the Flag Leaf and Developing Seed of Wheat ( Triticum Aestivum L.). BMC Genom..

[56] Wei B., Cai T., Zhang R., Li A., Huo N., Li S., Gu Y.Q., Vogel J., Jia J., Qi Y. (2009). Novel MicroRNAs Uncovered by Deep Sequencing of Small RNA Transcriptomes in Bread Wheat (Triticum Aestivum L.) and Brachypodium Distachyon (L.) Beauv. Funct. Integr. Genom..

[57] Chu Z., Chen J., Xu H., Dong Z., Chen F., Cui D. (2016). Identification and Comparative Analysis of MicroRNA in Wheat (Triticum Aestivum L.) Callus Derived from Mature and Immature Embryos during In Vitro Culture. Front. Plant Sci..

[58] Jin X., Jia L., Wang Y., Li B., Sun D., Chen X. (2020). Identification of Fusarium Graminearum-Responsive MiRNAs and Their Targets in Wheat by SRNA Sequencing and Degradome Analysis. Funct. Integr. Genom..

[59] Li Y.F., Wei K., Wang M., Wang L., Cui J., Zhang D., Guo J., Zhao M., Zheng Y. (2019). Identification and Temporal Expression Analysis of Conserved and Novel MicroRNAs in the Leaves of Winter Wheat Grown in the Field. Front. Genet..

[60] Liu H., Able A.J., Able J.A. (2021). Integrated Analysis of Small RNA, Transcriptome, and Degradome Sequencing Reveals the Water-Deficit and Heat Stress Response Network in Durum Wheat. Int. J. Mol. Sci..

[61] Ragupathy R., Ravichandran S., Mahdi M.S.R., Huang D., Reimer E., Domaratzki M., Cloutier S. (2016). Deep Sequencing of Wheat SRNA Transcriptome Reveals Distinct Temporal Expression Pattern of MiRNAs in Response to Heat, Light and UV. Sci. Rep..

[62] Liu H., Able A.J., Able J.A. (2020). Multi-Omics Analysis of Small RNA, Transcriptome, and Degradome in T. Turgidum—Regulatory Networks of Grain Development and Abiotic Stress Response. Int. J. Mol. Sci..

[63] Meng F., Liu H., Wang K., Liu L., Wang S., Zhao Y., Yin J. (2013). Development-Associated MicroRNAs in Grains of Wheat ( Triticum Aestivum L.). BMC Plant Biol..

[64] Noyszewski A.K., Ghavami F., Alnemer L.M., Soltani A., Gu Y.Q., Huo N., Meinhardt S., Kianian P.M.A., Kianian S.F. (2014). Accelerated Evolution of the Mitochondrial Genome in an Alloplasmic Line of Durum Wheat. BMC Genom..

