# Supplementary figures and images for: Wheat Long Noncoding RNAs from Organelle and Nuclear Genomes Carry Conserved microRNA Precursors Which May Together Comprise Intricate Networks in Insect Responses
Source: Int J Mol Sci. 2023 Jan 23;24(3):2226. doi: 10.3390/ijms24032226 (PMC9917100; doi:10.3390/ijms24032226)

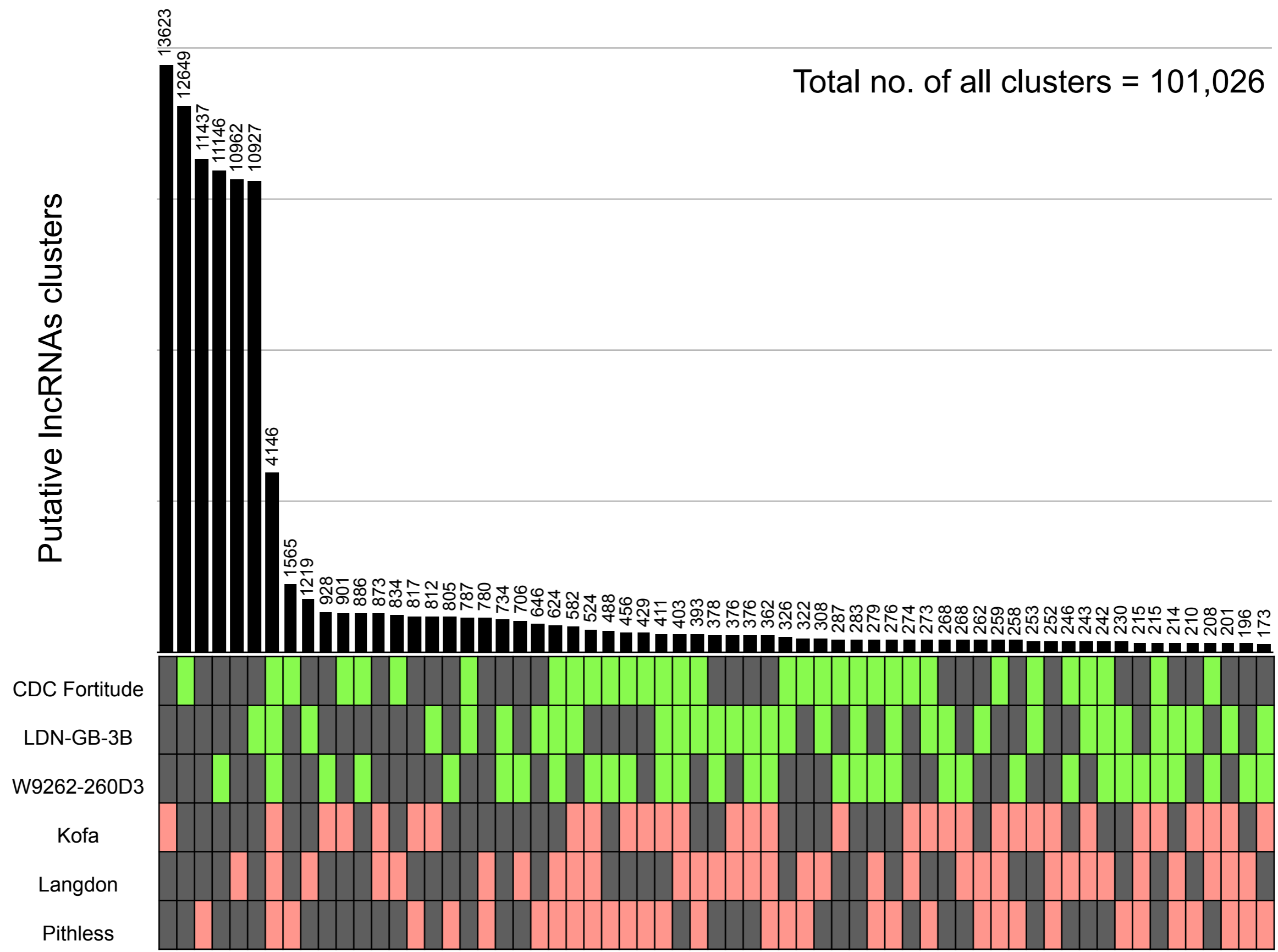

Supplement: Supplementary file 1 [file ijms-24-02226-s001.zip › lncRNA-MS-revisedSupplementaryFigureS1.pdf]

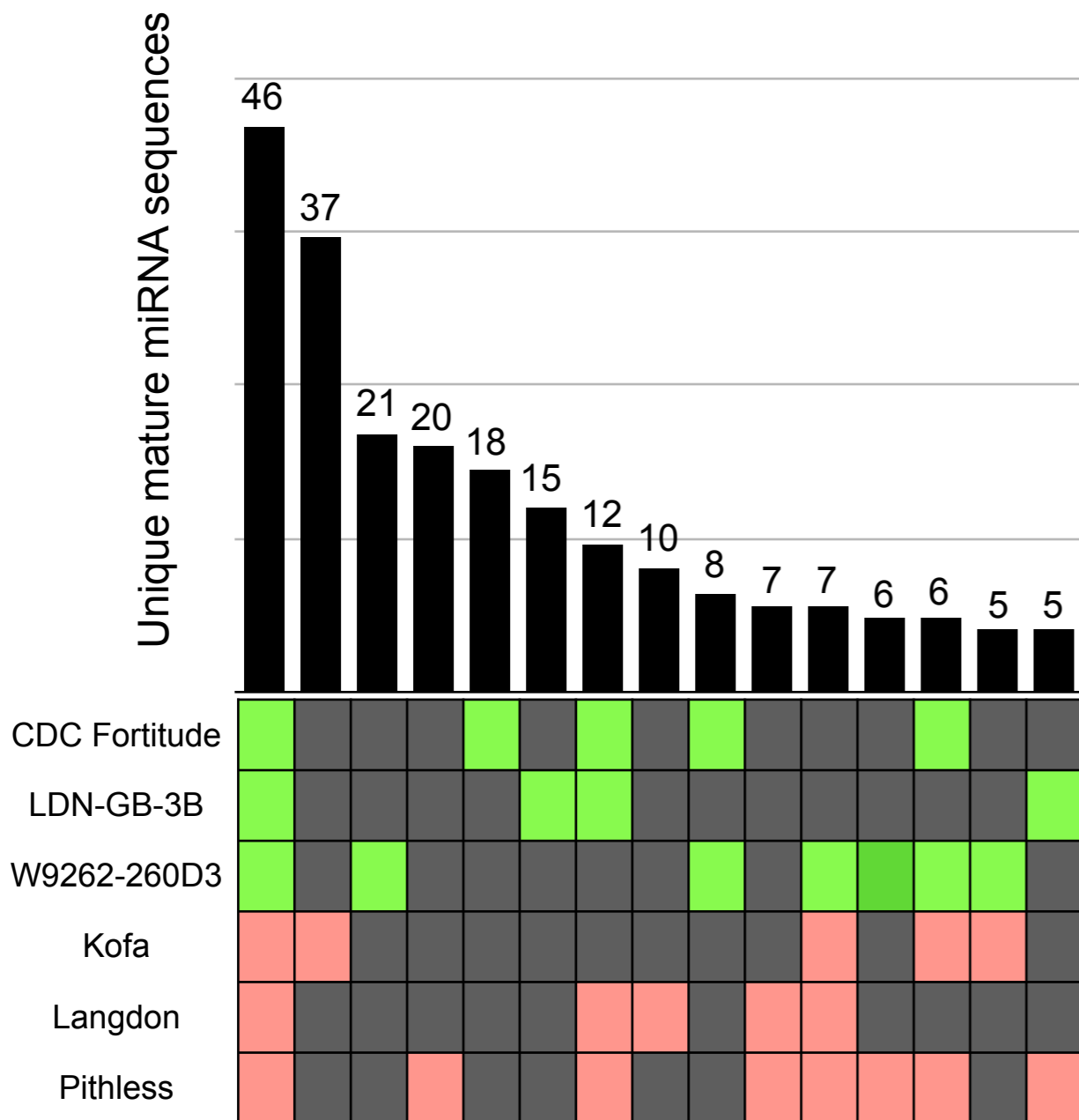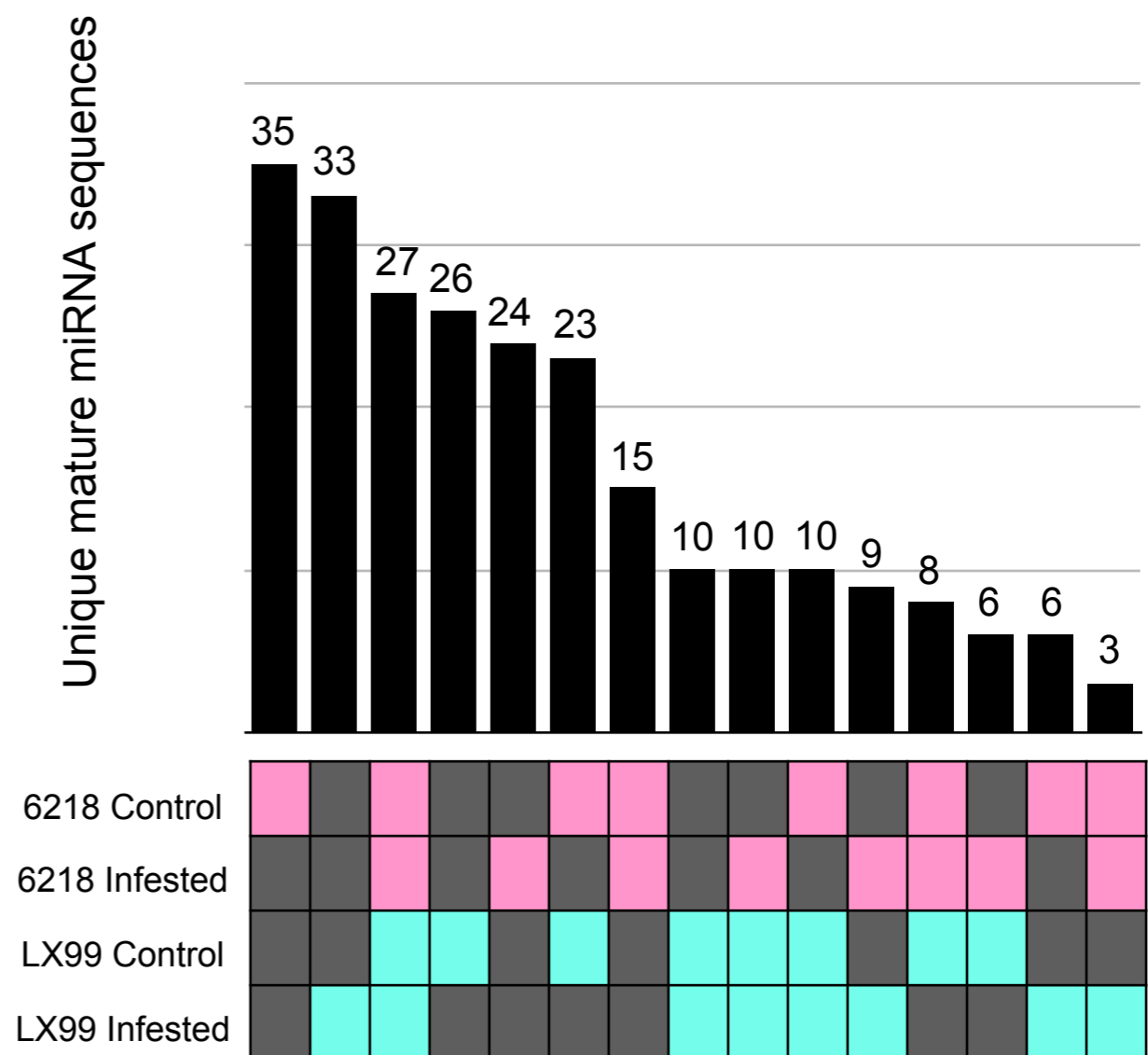

Supplement: Supplementary file 1 [file ijms-24-02226-s001.zip › lncRNA-MS-revisedSupplementaryFigureS2.pdf]
